# The glomerular filtration rate estimated by new and old equations as a predictor of important outcomes in elderly patients

**DOI:** 10.1186/1741-7015-12-27

**Published:** 2014-02-12

**Authors:** Gijs Van Pottelbergh, Bert Vaes, Wim Adriaensen, Cathy Matheï, Delphine Legrand, Pierre Wallemacq, Jean Marie Degryse

**Affiliations:** 1 Department of Public Health and Primary Care, Katholieke Universiteit Leuven, Kapucijnenvoer 33, blok J, PB 7001 3000, Leuven, Belgium; 2 Institute of Health and Society, Université Catholique de Louvain, Brussels, Belgium; 3 Laboratory of Analytical Biochemistry, Cliniques Universitaires St Luc, Université Catholique de Louvain, Brussels, Belgium

**Keywords:** Cardiovascular events, eGFR, Hospitalizations, Mortality, Renal function

## Abstract

**Background:**

The prevalence of chronic kidney disease (CKD) increases with age, and new glomerular filtration rate-estimating equations have recently been validated. The epidemiology of CKD in older individuals and the relationship between a low estimated glomerular filtration rate as calculated by these equations and adverse outcomes remains unknown.

**Methods:**

Data from the BELFRAIL study, a prospective, population-based cohort study of 539 individuals aged 80 years and older, were used. For every participant, five equations were used to calculate estimated glomerular filtration rate based on serum creatinine and/or cystatin C values: MDRD, CKD-EPIcreat, CKD-EPIcyst, CKD-EPIcreatcyst, and BIS equations. The outcomes analyzed included mortality combined with the necessity of new renal replacement therapy, severe cardiovascular events, and hospitalization.

**Results:**

During the follow-up period, which was an average of 2.9 years, 124 participants died, 7 required renal replacement therapy, 271 were hospitalized, and 73 had a severe cardiovascular event. The prevalence of estimated glomerular filtration rate values <60 mL/min/1.73 m^2^ differed depending on the equation used as follows: 44% (MDRD), 45% (CKD-EPIcreat), 75% (CKD-EPIcyst), 65% (CKD-EPIcreatcyst), and 80% (BIS). All of the glomerular filtration rate-estimating equations revealed that higher cardiovascular mortality was associated with lower estimated glomerular filtration rates and that higher probabilities of hospitalization were associated with estimated glomerular filtration rates <30 mL/min/1.73 m^2^. A lower estimated glomerular filtration rate did not predict a higher probability of severe cardiovascular events, except when using the CKD-EPIcyst equation. By calculating the net reclassification improvement, CKD-EPIcyst and CKD-EPIcreatcyst were shown to predict mortality (+25% and +18%) and severe cardiovascular events (+7% and +9%) with the highest accuracy. The BIS equation was less accurate in predicting mortality (-12%).

**Conclusion:**

Higher prevalence of CKD were found using the CKD-EPIcyst, CKD-EPIcreatcyst, and BIS equations compared with the MDRD and CKD-EPIcreat equations. The new CKD-EPIcreatcyst and CKD-EPIcyst equations appear to be better predictors of mortality and severe cardiovascular events.

## Background

Chronic kidney disease (CKD) is an important public health problem. First, dialysis and kidney transplantation impose a high cost on society. The cost of dialysis per patient per year in Belgium is more than 50,000 Euros, and >1% of the health budget of the Belgian government is used to cover dialysis costs. Second, patients with CKD have a high risk for cardiovascular events and mortality [1,2]. Therefore, many therapeutic and diagnostic drugs cannot be used or, if used, require dosing adaptation prior to use in patients with CKD.

The prevalence of CKD, when defined as an estimated glomerular filtration rate (eGFR) <60 mL/min/1.73 m^2^, increases with age. In Western countries [3,4], prevalence is approximately 10% at the age of 65 years and increases to 60% in individuals aged 80 years and older. The best method for estimating the GFR in older individuals remains unclear. Until recently, only limited validation of the equations used to estimate GFR in older individuals has been performed [5].

In 2012, three new GFR-estimating equations based on serum creatinine and serum cystatin C values, age, and gender were validated. Two of the studies were based on data from the CKD-EPI consortium [6] (with only limited numbers of older persons), and one was based on data from the Berlin Initiative Study (with only persons aged 70 and older) [7]. However, the epidemiology of CKD in older individuals and the relationship between low eGFR and adverse outcomes determined using these new equations have not been investigated.

In this study, we used the data from the BELFRAIL study to analyze the ability of GFR, estimated by older equations like the MDRD and CKD-EPI creatinine equations and the three new GFR equations, to predict mortality, necessity of renal replacement therapy (RRT), hospitalization, and severe cardiovascular events.

## Methods

### Study design

The BELFRAIL study is a prospective, observational, population-based cohort study of individuals aged 80 years and older in three well-circumscribed areas in Belgium. The study design and the characteristics of the cohort have previously been described in detail [8]. Briefly, 29 general practitioner (GP) centers were asked to recruit consecutive patients aged 80 years and older. Only three exclusion criteria were used: the presence of severe dementia, the necessity of palliative care, and medical urgency. The study protocol was approved by the Biomedical Ethics Committee of the Université Catholique de Louvain Medical School in Belgium (B40320084685), and all of the study participants provided informed consent.

The participants were recruited to the BELFRAIL study between 2 November 2008 and 15 September 15 2009. The GPs recorded the patients’ age, gender, and detailed medical history. The follow-up data regarding severe events in these participants were collected by questioning each participant’s GP 18 and 36 months after inclusion and baseline data collection. During this questioning, the following outcome parameters were collected: the exact date and cause of the total and cardiovascular mortality, severe cardiovascular events, necessity of RRT, and the date of and reason for hospitalizations.

### Laboratory tests

All blood samples were collected in the morning, and all measurements were performed in the laboratories of the Cliniques Universitaires St. Luc, Brussels. The serum concentration of creatinine was measured in the baseline blood sample using a UniCel DxC 800 Synchron instrument (Beckman Coulter, Inc., Brea, CA, USA). The creatinine assay was based on the Jaffé compensated isotope dilution mass spectrometry method, with total coefficient of variation ranging from 1.6% to 2% (105 to 1,049 μmol/L) in serum [9]. The N-latex cystatin C assay was based on an immunonephelometric method performed using the BNII analyzer from Siemens Diagnostics (Erlangen, Germany). The assay displayed total coefficient of variation from 2.3% to 4.3% (0.8 to 7.1 mg/L). The assay was run according to the manufacturer’s instructions and standards provided and met the new cystatin C International Federation of Clinical Chemistry and Laboratory Medicine standardization [10].

### Main parameters

Previously diagnosed hypertension, diabetes, myocardial infarction, cerebrovascular accident, and peripheral arterial disease, as well as past and current smoking history, were ascertained by each participant’s physician based on the medical files of the participant.

Five different equations were used to estimate the GFR, outlined below.

The isotope dilution mass spectrometry traceable MDRD equation (MDRD) [11]:

$$\begin{array}{l} \mathrm{GFR}=175\times {\left(S_{a}\right)}^{-1.154}\times {\left(\mathrm{Age}\right)}^{-0.203}\\ \;\times \left(0.742\;\mathrm{if}\;\mathrm{female}\right)\times \left(1.212\;\mathrm{if}\;\mathrm{black}\right) \end{array}$$

The Chronic Kidney Disease Epidemiology Collaboration equation [12] using creatinine (CKD-EPIcreat):

$$\begin{array}{l} \mathrm{GFR}=141\times \min{\left(S_{a}/k,l\right)}^{a}\times \max{\left(S_{a}/k,l\right)}^{-1,209}\\ \;\times 0.993^{\mathrm{Age}}\times \left(1.018\;\mathrm{if}\;\mathrm{female}\right)\\ \;\times \left(1.159\;\mathrm{if}\;\mathrm{black}\right) \end{array}$$

The Chronic Kidney Disease Epidemiology Collaboration cystatin C equation (CKD-EPIcyst) [6]:

$$\begin{array}{l} \mathrm{GFR}=133\times \min{\left(\mathrm{Scys}/0.8\right)}^{-0.499}\\ \;\times \max{\left(\mathrm{Scys}/0.8\right)}^{-1.328}\\ \;\times 0.996^{\mathrm{Age}}\left[\times 0.932\;\mathrm{if}\;\mathrm{female}\right] \end{array}$$

The Chronic Kidney Disease Epidemiology Collaboration creatinine and cystatin C equation (CKD-EPIcreatcyst) [6]:

$$\begin{array}{l} \mathrm{GFR}=135\times \min{\left(\mathrm{Scr}/k,l\right)}^{-a}\times \max{\left(\mathrm{Scr}/k,l\right)}^{-0.601}\\ \;\times \min{{\left(\mathrm{Scys}/0.8,1\right)}^{-}}^{.375}\\ \;\times \max{\left(\mathrm{Scys}/0.8,1\right)}^{-0.711}\\ \;\times 0.995^{\mathrm{age}}\left[\times 0.969\;\mathrm{if}\;\mathrm{female}\right]\left[\times 1.08\;\mathrm{if}\;\mathrm{black}\right] \end{array}$$

The Berlin Initiative Study Equation 2 (BIS) [7]:

$$\begin{array}{l} \mathrm{GFR}=767\times \mathrm{Scy}s^{-0.61}\times \mathrm{Sc}r^{-0.40}\\ \;\times \mathrm{ag}e^{0.57}\left[\times 0.87\;\mathrm{if}\;\mathrm{female}\right] \end{array}$$

The participants were classified into five categories based on their eGFR as follows: >90, 60 to 90, 45 to 60, 30 to 45, and <30 mL/min/1.73 m^2^.

### Statistical methods

Baseline differences between the groups with different eGFR values were assessed using the chi-square test for categorical parameters and one-way analysis of variance for normally distributed variables.

A Cox proportional hazards model was used to study the risk associated with the various CKD categories based on the different GFR-estimating equations for ‘renal death’ (defined as mortality or the necessity of RRT), cardiovascular mortality, severe cardiovascular events, and hospitalization. Two models were used. The first model (model 1) was adjusted for age and gender, and the second model (model 2) was adjusted for known risk factors (age, gender, hypertension, diabetes mellitus, history of a serious cardiovascular event, and smoking status). The odds ratios (ORs) for having no events (mortality, necessity of RRT, severe cardiovascular event, or hospitalization) during the follow-up period were estimated using logistic regression. The same models (models 1 and 2) were used to make adjustments. All of these analyses were performed using SPSS version 19.

The net reclassification improvement (NRI) [13], which was compared with the GFR estimated by the MDRD equation, was calculated with the other equations for the different outcomes and using a cutoff value of 60 mL/min/1.73 m^2^.

## Results

All of the necessary baseline data were available for all of the GFR calculations in 539 of the 567 participants in the BELFRAIL study. None of these 539 persons were lost to follow-up. The mean follow-up period from the baseline blood collection was 2.9 ±0.3 years. During this period, 124 of the participants died and 7 required RRT. Furthermore, 271 participants were hospitalized at least once, and 73 had at least one severe cardiovascular event. Table 1 lists the general characteristics of the population and the differences in these characteristics for participants with an eGFR >60 and <60 mL/min.

**Table 1:** General characteristics of the BELFRAIL population at baseline (n = 539), based on eGFR values at study entry, as estimated by different equations

	**All**	**MDRD <60 mL/min**	**CKD-EPIcreat <60 mL/min**	**CKD-EPIcyst <60 mL/min**	**CKD-EPIcreatcyst <60 mL/min**	**BIS <60 mL/min**
	**(n = 539)**	**(n = 237, 44%)**	**(n = 244, 45%)**	**(n = 405, 75%)**	**(n = 247, 46%)**	**(n = 431, 80%)**
Mean age	84.7	85.6^a^	85.7^a^	85.0^a^	84.7	84.7
	(SD 3.6)	(SD 4.0)	(SD 4.0)	(SD 3.8)	(SD 3.7)	(SD 3.7)
Male gender (%)	37	37	35	40	37	37
Hypertension (%)	70	73	79^a^	73	71	73
Diabetes mellitus (%)	19	20	21	19	19	20
History of myocardial infusion (%)	11	13	12	13	11	13
History of cerebrovascular accident (%)	8	9	8	9	9	9
History of peripheral arterial disease (%)	9	9	12	10	9	9
Smoker (%)	3	3	3	3	3	4

^a^*P* <0.05 compared with the subgroup of participants with eGFR >60 mL/min/1.73 m^2^. SD, standard deviation.

For the entire study population, the mean eGFR determined was 64 ±22 mL/min using the MDRD equation, 61 ±19 mL/min using the CKD-EPIcreat equation, 49 ±21 mL/min using the CKD-EPIcyst equation, 54 ±27 mL/min using the CKD-EPIcreatcyst equation, and 48 ±15 mL/min using the BIS equation. The prevalence of CKD defined as eGFR <60 mL/min differed based on the equation used and was as follows: 44% (MDRD), 45% (CKD-EPIcreat), 75% (CKD-EPIcyst), 65% (CKD-EPIcreatcyst), and 80% (BIS). The prevalence of severe CKD, defined as eGFR <30 mL/min, also differed as follows: 6% (MDRD), 7% (CKD-EPIcreat), 20% (CKD-EPIcyst), 13% (CKD-EPIcreatcyst), and 10% (BIS).

Table 2 shows the relationship between CKD stage and renal death (defined as mortality or the necessity of RRT), with participants with an eGFR of 60 to 90 mL/min as the reference group. The results are shown as hazard ratios (HRs) adjusted using two models with different confounders: age and gender; and age, gender, hypertension, diabetes mellitus, history of a serious cardiovascular event and smoking status. The absolute number of renal deaths was 43 (18%) in the MDRD reference group, 46 (16%) in the CKD-EPIcreat reference group, 13 (13%) in the CKD-EPIcyst reference group, 22 (14%) in the CKD-EPIcreatcyst reference group, and 13 (13%) in the BIS reference group.

**Table 2:** Relationship between estimated glomerular filtration rate and death or the necessity of renal replacement therapy

		**Estimated glomerular filtration rate (mL/min)**					
		**>90**	**60 to 90**	**45 to 60**	**30 to 45**	**<30**	***P***
MDRD	events	8/72	43/232	34/129	30/76	16/30	
	HR1	0.60	1	1.44	2.12	3.34	0.000
		(0.28 to 1.28)		(0.90 to 2.29)	(1.30 to 3.45)	(1.87 to 5.99)	
	HR2	0.60	1	1.35	2.03	3.32	0.000
		(0.28 to 1.28)		(0.85 to 2.15)	(1.25 to 3.30)	(1.82 to 6.05)	
CKD-EPIcreat	events	3/9	46/288	35/126	24/78	23/38	
	HR1	2.76	1	1.77	1.83	4.89	0.000
		(0.84 to 9.01)		(1.12 to 2.78)	(1.09 to 3.05)	(2.93 to 8.17)	
	HR2	3.01	1	1.65	1.72	5.04	0.000
		(0.91 to 9.96)		(1.05 to 2.61)	(1.03 to 2.88)	(2.95 to 8.60)	
CKD-EPIcyst	events	4/36	13/97	29/152	32/148	53/106	
	HR1	0.81	1	1.29	1.44	3.49	0.000
		(0.27 to 2.50)		(0.67 to 2.50)	(0.75 to 2.75)	(1.87 to 6.50)	
	HR2	0.76	1	1.19	1.43	3.41	0.000
		(0.25 to 2.36)		(0.61 to 2.32)	(0.75 to 2.75)	(1.81 to 6.42)	
CKD-EPIcreatcyst	events	6/37	22/154	30/157	34/123	39/68	
	HR1	1.23	1	1.34	1.82	4.14	0.000
		(0.49 to 3.04)		(0.77 to 2.35)	(1.05 to 3.17)	(2.39 to 7.15)	
	HR2	1.14	1	1.30	1.76	4.29	0.000
		(0.46 to 2.88)		(0.74 to 2.30)	(1.01 to 3.09)	(2.41 to 7.63)	
BIS	event/total	2/4	13/104	36/215	45/160	35/56	
	HR1	4.63	1	1.26	2.01	5.09	0.000
		(1.04 to 20.57)		(0.67 to 2.39)	(1.08 to 3.77)	(2.66 to 9.76)	
	HR2	4.75	1	1.31	1.98	5.58	0.000
		(1.06 to 21.25)		(0.69 to 2.50)	(1.05 to 2.50)	(2.84 to 10.96)	

HR1, Hazard ratio for model 1 - adjusted for age and gender; HR2, Hazard ratio for model 2 - adjusted for age, gender, hypertension, diabetes mellitus, history of a serious cardiovascular event and smoking status.

**Figure 1:**
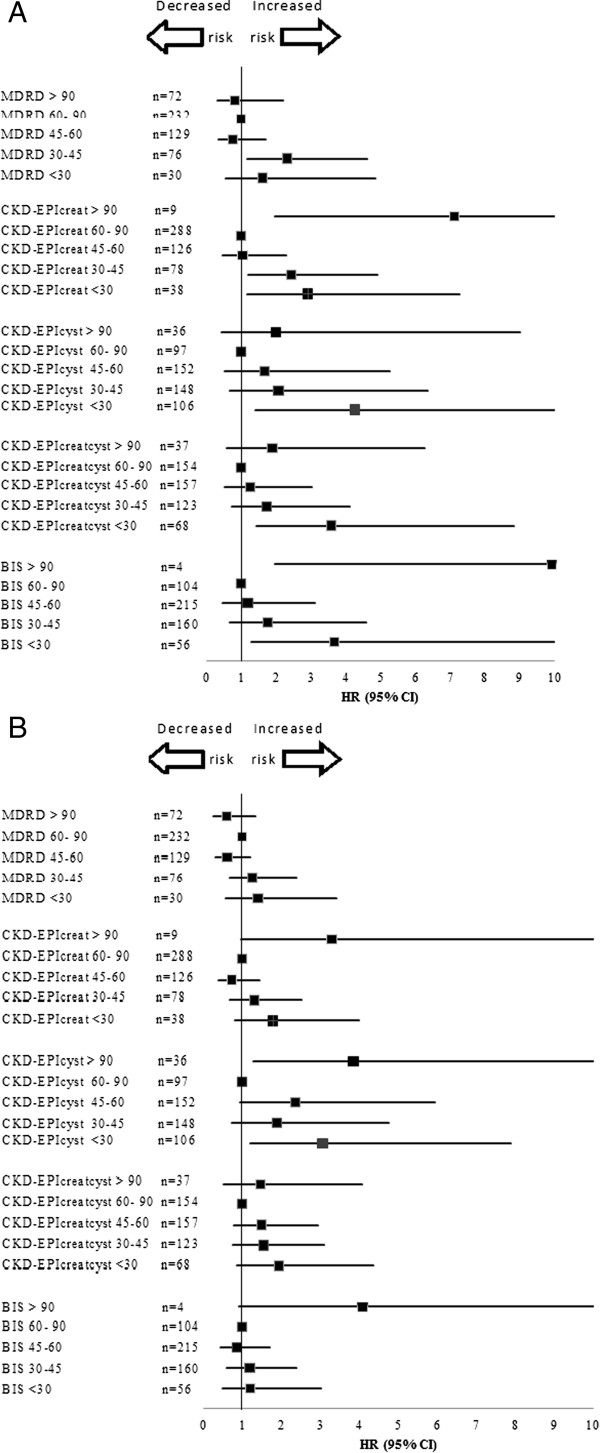
Cardiovascular mortality (A) and severe cardiovascular events (B) depending on the eGFR value estimated by different expressed as hazard ratios (HR) Values greater than 1.0 indicate an increased risk.

Significantly higher cardiovascular mortality was observed when the eGFR decreased in all five of the GFR-estimating equations (Figure 1A). By contrast (see Figure 1B), a lower eGFR did not predict a higher probability of severe cardiovascular events, except when the GFR was estimated by the CKD-EPIcyst equation. The relationship between the CKD-EPIcyst GFR and severe cardiovascular events appeared to be U-shaped, with more events occurring at higher eGFR values (eGFR >90 mL/min; adjusted HR of 3.85; 95% confidence interval (CI) 1.28, 11.64) and lower eGFR values (eGFR <30 mL/min; adjusted HR of 3.06; 95% CI 1.19, 7.90).

**Figure 2:**
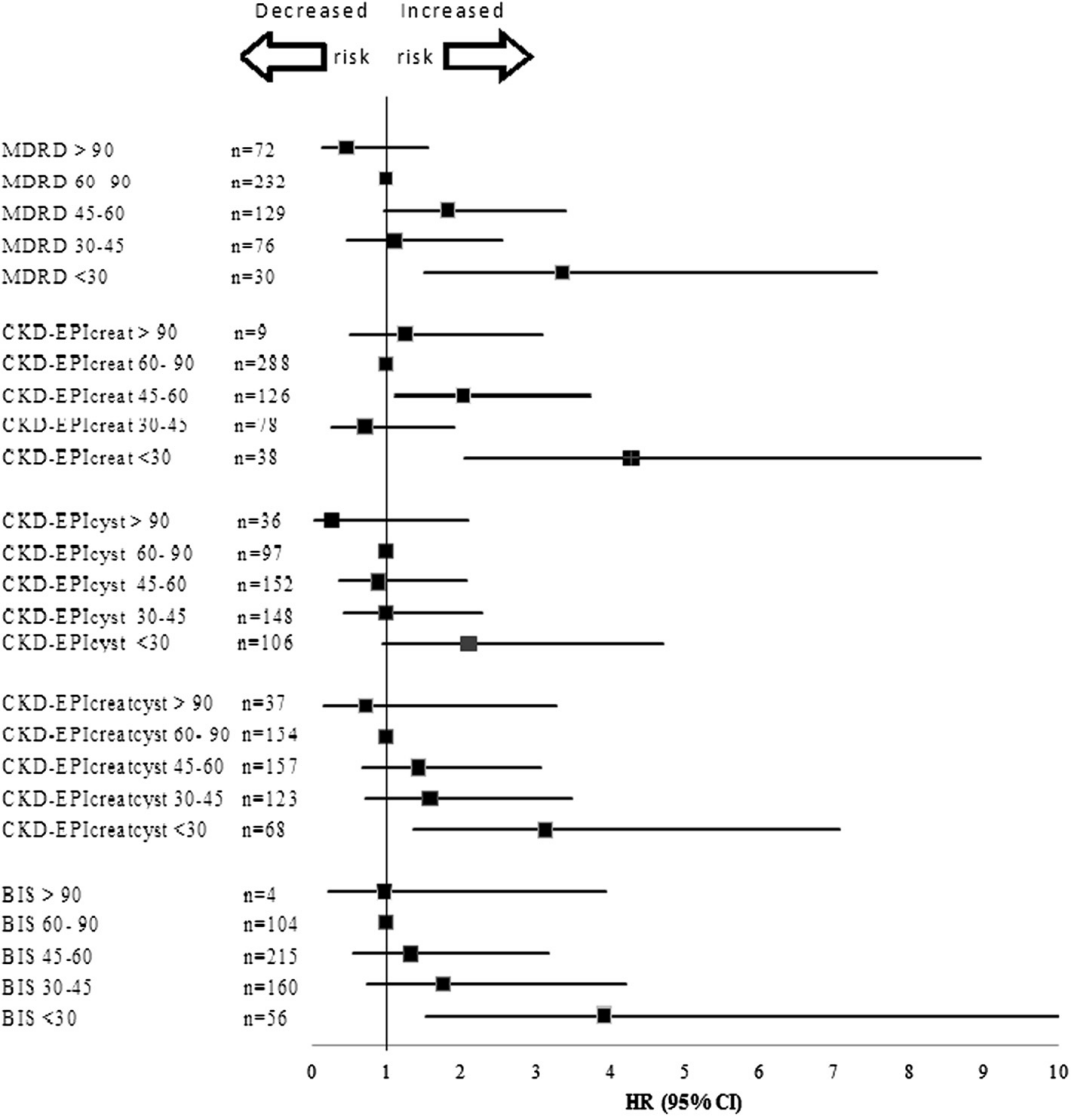
Hospitalizations depending on the eGFR value estimated by different expressed as hazard ratios (HR) Values greater than 1.0 indicate an increased risk.

Analysis of the interval to first hospitalization as a function of eGFR-based CKD stage (Figure 2) revealed that participants with an eGFR <30 mL/min had a higher risk for hospitalization, regardless of the GFR estimation equation used. Table 3 presents the probability of experiencing no events, as analyzed by linear regression analysis. The subgroup with an eGFR <30 mL/min had a higher risk of an event in all of the GFR estimations. All of the participants with an estimated GFR <60 mL/min based on the CKD-EPI had a higher risk of an event.

**Table 3:** Relationship between having no events during the 2.9-year follow-up period and the estimated glomerular filtration categories derived from logistic regression

		**Estimated glomerular filtration rate (mL/min)**					
		**>90**	**60 to 90**	**45 to 60**	**30 to 45**	**<30**	***P***
MDRD	events	38/72	110/232	49/129	22/76	6/30	
	OR1	1.17	1	0.76	0.55	0.32	0.003
		(0.69 to 1.99)		(0.49 to 1.19)	(0.31 to 0.98)	(0.13 to 0.83)	
	OR2	1.11	1	0.82	0.58	0.33	0.022
		(0.64 to 1.93)		(0.52 to 1.30)	(0.33 to 1.04)	(0.13 to 0.87)	
CKD-EPIcreat	event	4/9	143/288	45/126	27/78	6/38	
	OR1	0.73	1	0.64	0.65	0.23	0.02
		(0.19 to 2.82)		(0.41 to 1.00)	(0.38 to 1.12)	(0.09 to 0.56)	
	OR2	0.64	1	0.69	0.70	0.24	0.033
		(0.16 to 2.48)		(0.44 to 1.09)	(0.40 to 1.21)	(0.09 to 0.60)	
CKD-EPIcyst	event	20/36	54/97	65/152	60/148	26/106	
	OR1	1.00	1	0.59	0.56	0.31	0.000
		(0.46 to 2.17)		(0.35 to 0.99)	(0.33 to 0.95)	(0.17 to 0.58)	
	OR2	1.00	1	0.56	0.56	0.34	0.03
		(0.45 to 2.24)		(0.33 to 0.97)	(0.33 to 0.96)	(0.18 to 0.63)	
CKD-EPIcreatcyst	event	21/37	79/154	70/157	42/123	13/68	
	OR1	1.26	1	0.81	0.57	0.29	0.000
		(0.61 to 2.26)		(0.51 to 1.27)	(0.35 to 0.95)	(0.14 to 0.57)	
	OR2	1.41	1	0.83	0.63	0.32	0.03
		(0.66 to 3.00)		(0.52 to 1.32)	(0.38 to 1.06)	(0.15 to 0.65)	
BIS	event	2/4	54/104	103/215	55/160	11/56	
	OR1	1.02	1	0.90	0.59	0.30	0.002
		(0.14 to 7.64)		(0.56 to 1.44)	(0.35 to 0.80)	(0.14 to 0.64)	
	OR2	1.00	1	0.92	0.66	0.32	0.01
		(0.13 to 7.82)		(0.56 to 1.50)	(0.38 to 1.13)	(0.14 to 0.71)	

An event was defined as mortality, the necessity of renal replacement therapy, a serious cardiovascular event or hospitalization. OR1, Odds ratio adjusted for age and gender; OR2, odds ratio adjusted for age, gender, hypertension, diabetes mellitus, history of a serious cardiovascular event and smoking status.

With regard to absolute numbers, the CKD-EPIcys, CKD-EPIcreatcyst and BIS2 equations classified most of the participants who died in the subgroup with eGFR <60 mL/min at baseline (Table 2), but they only classified 33% (CKD-EPIcyst), 44% (CKD-EPIcreatcyst) and 25% (BIS) of the individuals experiencing no events in the group with eGFR >60 mL/min (see Table 3). The MDRD and CKD-EPIcreat equations not only predicted a higher absolute number of renal deaths in the group with eGFR >60 mL/min at baseline (see Table 2) but also higher numbers of renal deaths (66% with MDRD and 65% with CKD-EPI) in the group with eGFR <60 mL/min (Table 3).

The differences in the ability of the different GFR-estimating equations to predict adverse outcomes were further analyzed by measuring the NRI determined using the MDRD equation, using a cutoff value of 60 mL/min/1.73 m^2^. This NRI is reported in Table 4. The CKD-EPIcreat equation exhibited limited differences from the MDRD equation, and the BIS equation was less accurate than the MDRD equation in the prediction of renal death.

**Table 4:** **The net reclassification improvement generated by employing different formulas using MDRD as a reference and a cutoff value of 60 mL/min/1.73 m**^**2**^

**Equation used**	**Outcome**	**NRI using a 60 mL/min cutoff**	***P***
CKD-EPIcreat	Renal death	2%	0.08
	Cardiovascular events	0%	0.49
	Hospitalization	1%	0.13
	No events	−2%	0.05
CKD-EPIcyst	Renal death	25%	<0.01
	Cardiovascular events	7%	0.04
	Hospitalization	3%	0.30
	No events	1%	0.45
CKD-EPIcreatcyst	Renal death	18%	<0.01
	Cardiovascular events	9%	0.03
	Hospitalization	−2%	0.36
	No events	0%	0.49
BIS	Renal death	−12%	0.01
	Cardiovascular events	−2%	0.43
	Hospitalization	−7%	0.07
	No events	−7%	0.09

Renal death (mortality or renal replacement therapy), severe cardiovascular events, hospitalization or absence of events over a three-year period were used as outcomes. NRI, net reclassification improvement.

## Discussion

### Key findings

When using different equations to estimate the GFR, we found large differences (between 40% and 80%) in the prevalence of CKD (eGFR <60 mL/min) and large differences (between 6% and 20%) in the prevalence of severe CKD (eGFR <45 mL/min). Despite these differences in prevalence and regardless of the equation used, participants with an eGFR <30 mL/min were at extremely high risk for mortality, cardiovascular mortality and hospitalization. No relationship between eGFR and non-fatal cardiovascular events was found, except when the GFR was determined using the CKD-EPIcyst equation, which revealed a U-shaped relationship between eGFR and cardiovascular events. The MDRD and CKD-EPIcreat equations not only classified most of the participants with no events in the eGFR >60 mL/min group, but also higher numbers of participants with renal death in the same subgroup. The CKD-EPIcyst, CKD-EPIcreatcyst and BIS equations demonstrated the opposite pattern, identifying fewer renal deaths and classifying fewer numbers of participants with no events in the >60 mL/min subgroup. The NRI values suggest that the CKD-EPI cyst and CKD-EPIcreatcyst equations predict renal death and severe cardiovascular events more accurately than the other equations assessed. The BIS equation less accurately predicts renal deaths.

### Other literature

The CKD-EPIcyst, CKD-EPIcreatcyst and BIS equations are new. Consequently, only limited data exist regarding the use of these equations to determine the prevalence of CKD in older individuals. In the BIS validation study (individuals aged 70 years and older) [7], the mean eGFR of the study population was 8 and 10 mL/min higher when estimated by the CKD-EPI and MDRD equations, respectively, than when estimated by the BIS equation. In our study (individuals aged 80 years and older), this difference in mean eGFR was 13 mL/min (CKD-EPIcreat versus BIS) and 16 mL/min (MDRD versus BIS). Therefore, the mean difference in the mean eGFR obtained using these different equations appears to increase with age. To the best of our knowledge, no comparable data regarding the NRIs derived from the various GFR-estimating equations used in this article in older individuals have been reported.

It is not surprising that differences are observed since some of these equations use serum creatinine, others cystatin C, and some both to calculate the GFR. Creatinine is a breakdown product of creatinine phosphate in muscles. The generation of creatinine depends on the muscle mass, which probably explains racial, ethnic, sex- and age-related variation in the generation of creatinine. Creatinine is a breakdown product of meat, so dietary intake of meat is another source of variation in serum levels of creatinine. Thus, the serum level of creatinine is influenced by more than just the GFR. Cystatin C is a protein produced by all human cells with a nucleus. The generation of cystatin C is thought to be less variable than creatinine in and among individuals, but there is evidence that factors other than the GFR, like smoking, body mass index, inflammation, corticosteroid use, proteinuria, diabetes and race, have an influence on the cystatin C level. Cystatin C is also an better predictor than creatinine of cardiovascular events [14].

One of the main conclusions of our study is that an eGFR <30 mL/min is always related to a large increase in the risk of negative outcomes, such as mortality and hospitalization. This result is independent of the GFR-estimating equation used. It is less clear whether older individuals with an eGFR between 30 and 60 mL/min are all at increased risk for adverse outcomes. Previous studies regarding the risk for negative outcomes in subgroups of older individuals with eGFR values between 45 and 60 mL/min yielded contradictory results. Some studies [1,15] reported an increase in mortality, whereas other studies [2,16] reported a clear increase in mortality only when the eGFR was lower than 45 mL/min. These discrepancies may result from differences in the GFR-estimating equation used or differences in the study population. The latter explanation is especially likely because older individuals with CKD have lower relative risks for negative outcomes than younger individuals at the same stage of CKD [17-19]. Notably, in this context, the eGFR not only decreases over time but often also increases [17,18].

Another finding was the U-shaped relationship between the CKD-EPIcyst equation and mortality and cardiovascular events. The finding that people with higher eGFR values calculated based on cystatin C have more events needs to be researched further.

Given the high frequency of CKD in older individuals, it is important for physicians to distinguish between older patients with CKD who are at low risk for negative outcomes and older patients with CKD who are at high risk for negative outcomes. Various risk factors have been proposed for use in such a risk score, including the well-documented combination of eGFR and albuminuria [20-22], as well as the decrease in eGFR over time [23,24].

### Strengths and limitations

The main strength of our study is that the data originated from a population-based, prospective cohort study that has been demonstrated to be representative of the Belgian population [8]. Another strength is the employment of the correct standardization procedures for both creatinine and cystatin C. Furthermore, in addition to mortality and RRT, other relevant outcomes are reported, including hospitalizations, severe cardiovascular events and the probability of experiencing no events during a three-year period. The most important limitations of this study are the absence of a reference standard for measuring the true GFR and the measurement of albuminuria at baseline.

Finally, the eGFR cutoff value of 60 mL/min used to define CKD in older persons in this study is often debated since a part of the decline in renal function with aging could be due to physiological changes. However, there are many arguments for a decline in eGFR as a pathological process in most patients [25] and the internationally accepted eGFR cutoff to define CKD was used in this study.

## Conclusions

For octogenarians, a much higher prevalence of CKD and severe CKD was found when using the CKD-EPIcyst, CKD-EPIcreatcyst and BIS equations compared with the MDRD and CKD-EPIcreat equations. The CKD-EPI creatinine equation performed similarly to the MDRD equation in predicting adverse outcomes. The new CKD-EPIcreatcyst and CKD-EPIcyst equations appeared to better predict mortality or RRT and severe cardiovascular events. By contrast, the new BIS equation was less accurate at predicting mortality and RRT compared with the MDRD equation.

## Abbreviations

BIS: The Berlin Initiative Study Equation 2; CI: confidence interval; CKD: chronic kidney disease; CKD-EPIcreat: The Chronic Kidney Disease Epidemiology Collaboration equation using creatinine; CKD-EPIcreatcys: The Chronic Kidney Disease Epidemiology Collaboration creatinine and cystatin C equation; CKD-EPIcyst: The Chronic Kidney Disease Epidemiology Collaboration cystatin C equation; eGFR: estimated glomerular filtration rate; GFR: glomerular filtration rate; GP: general practitioner; HR: hazard ratio; MDRD: The isotope dilution mass spectrometry traceable equation; NRI: net reclassification improvement; OR: odds ratio; RRT: renal replacement therapy.

## Competing interests

The authors declare that they have no competing interests.

## Authors’ contribution

GVP conducted the statistical analyses, drafted the manuscript, and all of the authors made critical revisions for important intellectual content. All of the authors contributed to the analysis and interpretation of the data. BV, JDG and PW contributed to the study concept and design and obtained funding for the study. JDG supervised the study. All authors read and approved the final manuscript.

## Authors’ information

GVP is a Fellow of the Research Foundation Flanders.

## Pre-publication history

The pre-publication history for this paper can be accessed here:

http://www.biomedcentral.com/1741-7015/12/27/prepub
